# Myocardial contractility indices based on strain imaging

**DOI:** 10.1186/1532-429X-16-S1-P333

**Published:** 2014-01-16

**Authors:** Tazim Merchant, Danielle Janosevic, Meghana Jayam, Madhavi Kadiyala, Simcha Pollack, Jie J Cao, Nathaniel Reichek

**Affiliations:** 1 St. Francis Hospital,Research and Education Foundation, Roslyn, New York, USA; 2 Cardiology, Stony Brook University, Stony Brook, New York, USA

## Background

The principal determinants of chronic left ventricular(LV) dysfunction are reduced myocardial contractility and afterload excess due to adverse LV remodeling. To determine the relative contribution of each to a given instance of LV dysfunction, reliable quantitative indices of both myocardial contractility and afterload are needed. At the LV chamber level, ventricular volume and LV pressure can be used in a ventricular elastance model. But at the myocardial level, afterload must be normalized per unit of myocardium, conventionally done using wall stress (WS) calculations, while myocardial function is best characterized as systolic myocardial deformation, or strain (ST). Prior experimental model studies have suggested that the ratio of strain to afterload may be an effective contractility index. However, this has not been evaluated in human disease. We have recently shown that a nongeometric LV end-systolic afterload index (NGI, = (end-systolic LV pressure(P) × volume(V))/LV mass(M), or PV/M), may be superior to conventional circumferential WS (CWS) as a quantitative measure of afterload at the myocardial level, and correlates more closely than CWS with circumferential ST(CST), Therefore, we evaluated the ratios CST/CWS and CST/PV/M, as candidate contractility indices in normals(NL) and in patients with nonischemic dilated cardiomyopathy(CM).

## Methods

In NLs (n = 39,46% women, age 54.6 +14.6 yrs) and CM(n = 35,23% women, age 50.8+5.0 yrs) we obtained breathhold volumetric CMR cines, SPAMM tagged cines and cuff systolic blood pressure and derived EF, global circumferential strain(CST) and mean strain rate(CSR), using feature-tracking(FT) ST, (TomTec Imaging Systems) and in a subset, HARP ST(Diagnosoft). End-systolic stress(CWS,(Mirsky, Biophys. J.1969)) and PV/M were also determined.

## Results

(Table 1) EF, CST and CSR were markedly reduced in CM and CWS and PV/M markedly elevated, consistent with afterload excess. However, the CST/CWS and CST/PV/M ratios were also markedly reduced in CM, indicative of contractile depression, with generally strong correlations of these ratios with EF and CSR, particularly in CM(Table 2). In stepwise regression, FT CST/CWS and CST/PV/M ratios were the principal correlates of LV EF, not absolute afterload. AUCs for FT CST/CWS and CST/PV/M ratios against EF exceeded 0.90 (p < 0.0001)

**Table 1:** 

FT	CM(n = 35)	NL(n = 39)	p
EF	27.2 ± 10.8%	58.4 ± 4.6%	< 0.0001

CST	-10.7 ± 5.3%	-23.9 ± 4.3%	< 0.0001

CSR %/sec	-32.1 ± 14.8%	-65.7 ± 14.9	< 0.0001

CWSx10(3)dyn/cm(2)	307.6 ± 9.2	176.2 ± 42.1	< 0.0001

PV/M mm Hg	162.6 ± 8.9	84.4 ± 8.4	< 0.0001

CST/CWS %/10(3)dyn/cm(2)	-0.039 ± 0.025	-0.145 ± 0.053	< 0.0001

CST/PV/M mmHg	-0.079 ± 0.062	-0.301 ± 0.114	< 0.0001

			

HARP			p

EF	CM (n = 11)	NL(n = 38)	< 0.0001

CST	-8.4 ± 2.6	-17.4 ± 2.3	< 0.0001

CSR %/sec	-26.2 ± 8.0	-47.6 ± 6.5	< 0.0001

CWSx10(3)dyn/cm(2)	272.9 ± 114.0	174.7 ± 41.6	0.017

PV/M mm Hg	141.09 ± 60.5	83.3 ± 17.3	0.010

CST/CWS %/10(3)dyn/cm(2)	-0.034 ± 0.015	-0.106 ± 0.032	< 0.0001

CST/PV/M mmHg	-0.071 ± 0.044	-0.217 ± 0.052	< 0.0001

**Table 2:** Strain/Afterload Ratios Versus EF and Strain Rate

FT	n	Spearman r	p
CST/CWS vs. EF NL	39	-0.51	0.0009

CST/PV/M vs. EF NL	39	-0.70	< 0.0001

CST/CWS vs. EF CM	35	-0.87	< 0.0001

CST/PV/M vs. EF CM	35	-0.88	< 0.0001

CST/CWS vs. CSR NL	39	0.68	< 0.0001

CST/PV/M vs. CSR NL	39	0.69	< 0.0001

CST/CWS vs. CSR CM	35	0.86	< 0.0001

CST/PV/M vs. CSR CM	35	0.90	< 0.0001

HARP			

CST/CWS vs. EF NL	38	-0.2	ns

CST/PV/M vs. EF NL	38	-0.54	0.0005

CST/CWS vs. EF CM	11	-0.72	0.013

CST/PV/M vs. EF CM	11	-0.80	0.003

CST/CWS vs. CSR NL	38	0.54	0.0005

CST/PV/M vs. CSR NL	38	0.52	0.0008

CST/CWS vs. CSR CM	11	0.55	ns

CST/PV/M vs. CSR CM	11	0.66	0.026

## Conclusions

: Strain/stress and strain/PV/M ratios are promising noninvasive myocardial contractile indices which can depict the contribution of contractile depression to reduced myocardial function. However, demonstration of the sensitivity of these indices to changes in inotropic state are also needed to validate these measures for potential research and clinical applications.

## Funding

St. Francis Research Foundation.

